# Mitral regurgitation detection and central/eccentric classification using transformer‐based deep learning in multi‐view echocardiography

**DOI:** 10.1002/acm2.70584

**Published:** 2026-04-20

**Authors:** Xiaofang Zhong, Jiancheng Zhang, Yuanyuan Sheng, Ying Guo, Yumei Yang, Yuxiang Huang, Hongjuan Zhao, Wei Zeng, Lixin Chen, Dong Ni, Jinfeng Xu, Wufeng Xue, Yingying Liu

**Affiliations:** ^1^ Department of Ultrasound, Shenzhen People's Hospital (The First Affiliated Hospital, Southern University of Science and Technology; The Second Clinical Medical College, Jinan University) Shenzhen China; ^2^ The National‐Regional Key Technology Engineering Laboratory for Medical Ultrasound, School of Biomedical Engineering, Health Science Center Shenzhen University Shenzhen China; ^3^ Medical Ultrasound Image Computing (MUSIC) Laboratory Shenzhen University Shenzhen China; ^4^ Marshall Laboratory of Biomedical Engineering Shenzhen University Shenzhen China

**Keywords:** central, eccentric, mitral regurgitation, multi‐view, transformer

## Abstract

**Background:**

Fully automated diagnostic echocardiography allows for broader screening, earlier diagnosis for patients with mitral regurgitation (MR). This feasibility study developed a deep learning (DL) framework to automatically detect MR in echocardiography videos and classify regurgitation samples into central or eccentric regurgitation.

**Methods:**

We designed a Transformer‐based deep learning model for fully automatic Doppler video detection of MR. This framework can automatically detect MR and classify central or eccentric regurgitation. The algorithm was trained, validated, and tested using retrospectively selected studies. A prospective dataset of 217 patients was used as an independent test database.

**Results:**

(a) The model demonstrated exceptional diagnostic accuracy for MR, achieving accuracy rates of 0.94(95%CI: 0.90‐0.97) and 0.92(95%CI: 0.89‐0.96) in retrospective and prospective test sets, respectively, with area under the curve (AUC) values of 0.98 (95%CI: 0.96‐1.0) and 0.97 (95%CI: 0.95‐0.99). Its performance aligned with that of middle seniority physicians. (b) The model achieved an accuracy of 0.93 in identifying central and eccentric MR across both retrospective and prospective test sets, AUC values of 0.96 (95%CI: 0.89‐0.99) and 0.95 (95%CI: 0.89‐0.98), respectively. (c) On the tasks of MR, the diagnostic performance of the model in multiple views was better than that of the parasternal long‐axis view (PLAX) alone or the apical four‐chamber view (A4C) in both retrospective and prospective datasets.

**Conclusion:**

The Transformer‐based algorithm proposed in this study can automate and improve the efficiency of clinical workflows, screen multi‐view for the presence of MR, and assist in classifying regurgitation characteristics.

## 1 INTRODUCTION

Mitral regurgitation (MR) is one of the most common heart valve diseases. According to statistics, among people older than 75 years, the incidence of moderate to severe MR can reach 9.3%.^1^ Among valvular heart diseases, approximately 12.5% are affected.^2^ High mortality rate, frequent occurrence of heart failure, and low surgical treatment rate are the key challenges in the current management of MR.^3^ Due to compensatory dilation of the ventricle, patients with mitral regurgitation (MR) can be asymptomatic for many years. Once the decompensation phase progresses, irreversible left ventricular dysfunction occurs. If early intervention is performed, the survival rate can significantly improve, thereby benefiting the patient and reducing the burden on the patient's family and socioeconomic status.

Echocardiography is the preferred imaging method for diagnosing MR, and early detection of pathological MR may be important for monitoring and treating this disease.^4^ Echocardiography has the advantages of easy operation, low cost, wide application range, and noninvasive. With the advancements in technology, image quality and portability have continuously improved, and the costs related to image acquisition have decreased. These factors have led to a massive surge in the number of examinations, which strains the limited ability of qualified physicians to provide accurate interpretations in a timely manner. The interpretation of the examinations also relies on the operator's experience, which may lead to misdiagnosis and significant inter‐observer variability (coefficient of variation as high as 72%),^5^ especially in eccentric MR patients with unique hemodynamic characteristics. In addition, the mitral valve often has organic lesions in eccentric regurgitation, so the accurate identification of whether it is eccentric regurgitation has important reference value for clinical judgment of the mechanism of regurgitation. Developing artificial intelligence (AI) has provided useful reference information for the assessment of MR. In recent years, visual Transformer^6^ have been widely used and have made great progress in interpreting echocardiographic research, including automatic image quality assessment, view classification, segmentation, volume and ejection fraction calculation, and disease classification. The Swin Transformer^7^ is a deep learning (DL) model based on the Transformer architecture that is both efficient and flexible when processing visual information and can capture a variety of features from fine details to a broader context. This approach improves the computational efficiency and model performance.

Clinical diagnosing MR typically incorporates comprehensive evaluation of data from multiple views, but frequently leads to labor‐intensive and time‐consuming procedures. Most current methods are based on a single view image or video and cannot effectively utilize multi‐view video information.^8^, ^9^, ^10^, ^11^, ^12^Most current methods are based on single or multiple static images and cannot effectively utilize the video information of multi‐view.^8^, ^9^, ^10^, ^11^ Integration of videos from different views has proven to improve accuracy in assessing the severity of heart valve regurgitation.^13^ Moreover, there is limited research on applying the Transformer in echocardiographic image analysis for diagnosing MR. The study aimed to explore the feasibility of developing a Transformer‐based DL framework for automating multi‐view echocardiography video analysis. This framework can detect the presence of MR and identify central or eccentric MR, thus serving as a screening tool to assist doctors in enhancing their diagnostic capabilities.

## 2 METHODS

### 2.1 Database creation

The study involved algorithm development and initial testing on a retrospective dataset and final testing on a prospective, real‐world dataset from a consecutively collected echocardiographic study, as summarized in Figure S1. Outpatients and inpatients who underwent examinations from September 2018 to September 2022 were retrospectively included. The exclusion criteria for patients were as follows: < 18 years of age, poor image quality such as cardiac structure was unclear, the view was very non‐standard, severe view loss, no simultaneous connection to an electrocardiogram, a history of mitral valve surgery and cases with multiple jets of regurgitation, unclear distinctions between central and eccentric regurgitation. Finally, 733 patients were included in this study (no MR = 241, MR = 492; central MR = 343, eccentric MR = 149), and a total of 3405 videos were analyzed. Among the initial cases available for analysis, the following views were used for model training: color Doppler parasternal long‐axis view (PLAX‐Color), color Doppler apical four‐chamber view (A4C‐Color), color Doppler apical view three‐chamber view (A3C‐Color), color Doppler apical two‐chamber view (A2C‐Color) and color Doppler apical five‐chamber view (A5C‐Color).

The prospective testing dataset included data obtained between October 2022 and August 2023. The test dataset included 217 outpatients and inpatients (no MR = 70, MR = 147; central MR = 106, eccentric MR = 41) for a total of 1211 videos.

### 2.2 Instruments

Different models of Philips color Doppler ultrasound diagnostic instruments and adult heart probes were used. Image acquisition and parameter measurement: Patients were scanned in the left decubitus position while calmly breathing while connected to the synchronous electrocardiogram, and the frame rates were set at 51–70 frames/second, and the color scale was 60–70 cm/s. The videos of PLAX‐Color, A4C‐Color, A3C‐Color,A2C‐Color and A5C‐Color for four consecutive cardiac cycles with low heart rate variability were saved in DICOM format.

The degree of MR was judged according to the recommendations of the American Society of Echocardiography (ASE) guidelines and was divided into mild, moderate, and severe.^4^ Mild: small central jet area<20% LA, on Doppler, Small vena contracta < 0.3 cm; Moderate: Central jet MR 20%–50% LA of late systolic eccentric jet MR; Vena contracta<0.7 cm; Regurgitation volume < 60 mL; effective regurgitation orifice area (EROA)<0.40 cm^2^;Severe: Central jet MR > 50% LA or holosystolic eccentric jet MR; Vena contract≥7 cm; Regurgitation volume ≥60 mL; EROA≥0.40 cm^2^.

### 2.3 MR automatic diagnosis pipeline

Our method has two steps: (a) MR detection, which detects whether there is MR from the input color Doppler ultrasound videos of any multi‐view, and (b) central or eccentric MR classification, which is useful for predicting MR. For MR cases, central or eccentric MR is determined from the input multi‐view videos. Inspired by the Swin Transformer,^7^ lightweight Transformer,^11^ and multiple instance learning,^14^, ^15^, ^16^ two Swin Transformer‐based models were developed with hyperparameter tuning.

### 2.4 Echocardiographic data preprocessing and labeling

We used Python (version 3.8) and the Pydicom library to convert the original DICOM images into AVI format and applied the public data processing pair platform for label setting and annotation. Since the sample volume is unchanged throughout the entire video, we use the Open CV library to automatically extract the region of interest (ROI) area of the sample volume in the first frame of the color Doppler video and then eliminate patient information and other irrelevant data (heart rate, electrocardiogram curve, etc.). Furthermore, all videos are acquired at a uniform resolution of 800×600. To balance computational efficiency with the preservation of cardiac anatomical integrity, a square ROI centered on the heart is extracted from each frame and resized to 224×224 pixels. This standardized input size aligns with the most widely adopted resolution in DL frameworks, ensuring seamless compatibility with pretrained model architectures while maintaining structural fidelity during preprocessing. Each video segment is obtained by randomly sampling 10 frames online. The final input of the MR detection and classification model is (*n*, 10, 224, 224), where n is the number of videos. The echocardiographic data included the PLAX‐Color, A4C‐Color, A3C‐Color, A2C‐Color, and A5C‐Color dynamic video images. Most videos of the same view had a score ≥1, some views were missing, and there was an imbalance of aspects. To overcome the difficulty of obtaining an unbalanced data distribution in the training dataset, we used an online random sampling method when the samples were limited. During training, we randomly sample 10 consecutive frames from each video to augment data diversity. In the testing phase we extract three nonoverlapping 10‐frame clips sequentially from the start to cover the video broadly, then aggregate their predictions for the final result. Multiple videos can be combined to achieve data enhancement. The training, validation, and test sets were divided according to the patients at a ratio of 7:1:2. Furthermore, all videos from the same patient were grouped into the same set, ensuring that there was no overlap between patients across the training, validation, and test sets, thus preventing data leakage. The training set image annotation was completed by 1 of 5 rigorously trained and experienced annotation doctors, The test set was annotated one by one by 6 untrained doctors with different experience levels. The 6 labeled doctors included 2 experts (> 20 years of experience), 2 mid‐level doctors (working experience 5–7 years), and 2 junior doctors (working experience 1–2 years). The criteria for determining the presence of MR and its classification as central or eccentric MR were based on the review in echocardiographic videos by two experts. In cases of disagreement, a third expert was consulted to make the final determination.

MR is detectable only in specific color Doppler views during systole. Classification as central or eccentric is based on jet morphology and spatial location. Central MR features a jet emerging centrally from the mitral orifice and spreading symmetrically in the left atrium, while eccentric MR appears as a jet deviating to one side, flowing adjacent to the wall. Manual marking: degree of MR (no MR, mild MR, moderate MR, or severe MR); MR phase (start frame, maximum frame, or end frame); central or eccentric regurgitation. These annotations served as ground truth to supervise the training of two multi‐view Transformer models. The first model detects regurgitation across multiple views, while the second classifies the eccentricity of regurgitation based on the shape of the jet and its spatial position. The models take video clips as input, with each clip containing a full cardiac cycle from a standard view.

### 2.5 **Building a** DL **model**

**FIGURE 1:**
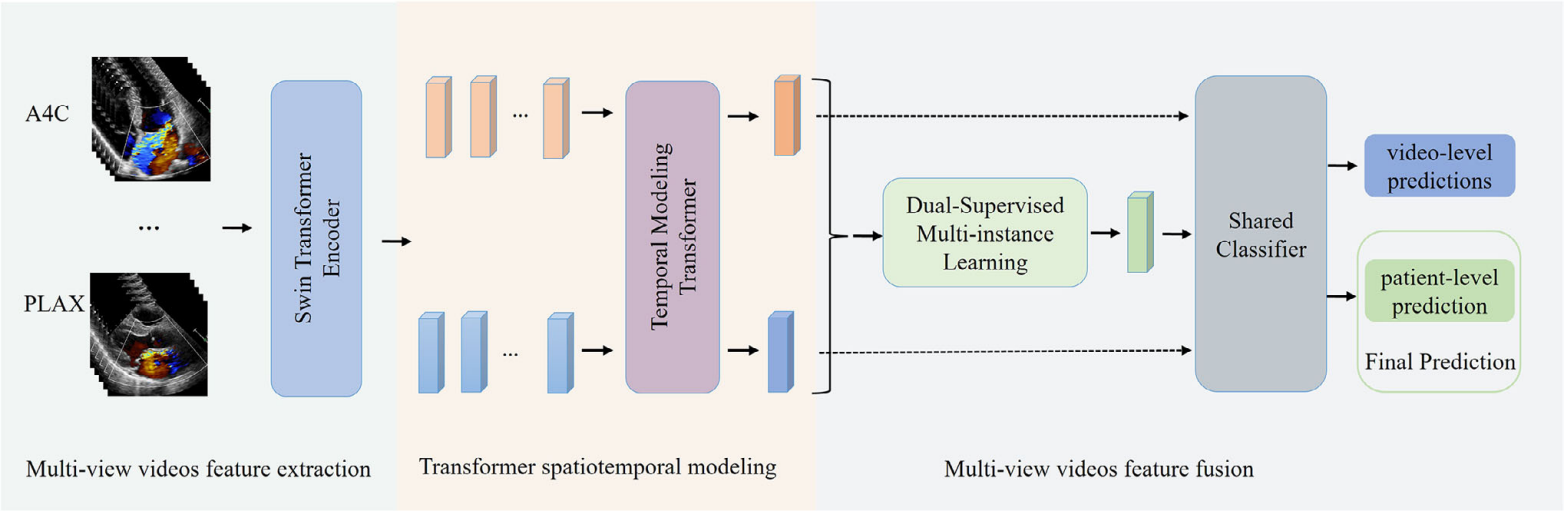
Workflow of MR detection and centrality or eccentricity MR classification systems (same model framework).

Our DL model workflow is shown in Figure 1, comprises three core modules: a Swin Transformer Encoder, a Transformer based Spatiotemporal modeling module, and a Dual‐Supervised Multi‐Instance Learning (DS‐MIL).

Swin Transformer Encoder: Each frame from the multi‐view color Doppler video is processed by a shared Swin Transformer encoder to derive high level spatial representations. The encoder's spatial heatmap highlights regions of interest per frame, enabling clinicians to confirm that model decisions rely on anatomically meaningful signals rather than artifacts or background noise.

Transformer based Spatiotemporal Modeling: With frame level features extracted, the Transformer's temporal modeling strength captures dynamic patterns video segments. To enhance training effectiveness and drawing inspiration from the literature,^16^ we introduce self‐attention matrix constraint (SSA) based on the attention matrix to supervise the learning of the Transformer module: (a) approximate matrix symmetry and (b) low information entropy. This module also produces a temporal attention heatmap, indicating which cardiac frames the model deems most informative.

Dual‐Supervised Multi‐Instance Learning (DS‐MIL): This module adaptively fuses features from multiple views, aggregating 1 to n available views into a unified patient level representation. Its design is inspired by clinical logic: if MR appears in any standard view, the diagnosis is MR; if eccentric MR appears in any view, the diagnosis is eccentric MR. Max pooling fuses video level features while accommodating incomplete views, improving clinical utility.

Moreover, MR detection and central or eccentric regurgitation classification use the same model architecture, which also illustrates the versatility of our model in handling multi‐view tasks. For the detailed module architecture, please refer to the supplementary materials and Figures S2–S4.

### 2.6 Model performance and statistical analysis

Regarding the detection of MR presence, the DL classification model was evaluated against the ground truth. The evaluation metrics include accuracy, precision, recall, and F1 score,^17^ confusion matrix and the area under the receiver operating characteristic (ROC) curve. Performance metrics are reported as the mean and 95% confidence intervals (CIs) derived from1000 bootstrap iterations.

We used class activation map technology which allows visualization of model performance by comparing actual (true label) versus predicted class.

To evaluate the performance of the model for detecting MR, we compared the metrics, including accuracy, precision, recall, and F1 score, to those of 6 physician echocardiographers with different years of experience. For this analysis, physicians were provided with the same images as those provided for the DL algorithm.

In addition, we evaluated the advantages of the algorithm for multi‐view MR detection and central and eccentric MR classification. We also compared the multi‐view accuracy, precision, recall, F1 score and AUC with the PLAX‐Color and A4C‐Color results.

The results were analyzed using algorithms written in Python (version 3.8) and PyTorch (version 1.8). Continuous variables are expressed as the mean ± standard deviation, median and interquartile range (IQR), or count and percentage.

## 3 RESULTS

### 3.1 Basic clinical and echocardiography data distribution

Table 1 and Table 2 summarize the basic clinical characteristics, echocardiography characteristics, and distribution of the datasets of the patients retrospectively included in the study. Tables 3 and 4 summarize the basic clinical characteristics, echocardiography characteristics, and distribution of the datasets of the prospectively included study patients Table S1–S4 provides the datasets used for MR detection and central or eccentric regurgitation classification with different patients, view videos, and frame rates.

**TABLE 1:** **Clinical** characteristics of the retrospective database.

	No MR(*N *= 241)		MR(*N *= 492)	
	Training/Validation	Test	Training/Validation	Test
	(*N *= 200)	(*N *= 50)	(*N *= 387)	(*N *= 96)
Age(*y*)	42.76 ± 14.24	46.12 ± 13.80	53.28 ± 16.42	53.28 ± 14.79
Male(*n*)	114(57%)	29(58.00%)	215(55.56%)	58(60.41%)
Height(cm)	165.08 ± 7.55	164.88 ± 8.91	163.93 ± 8.20	164.24 ± 8.13
Weight(kg)	62.07 ± 10.00	65.16 ± 14.60	63(53‐68.50)	61.85 ± 11.18
BSA(*m* ^2^)	1.67 ± 0.17	1.71 ± 0.23	1.66 ± 0.26	1.66 ± 0.18
HR(bpm)	77.52 ± 12.23	71.86 ± 11.68	78(67‐86)	78.36 ± 17.10
Comorbidities(*n*,%)				
Hypertension	60(30.00%)	14(28%)	97(25.06%)	35(36.45%)
Diabetes	51(25.50%)	12(24%)	102(26.36%)	29(30.20%)
Atrial fibrillation	0(0)	0(0)	52(13.43%)	10(10.41%)
Heart failure	0(0)	0(0)	57(14.72%)	12(12.5%)
RHD	1(0.50%)	0(0)	38(9.82%)	8(8.33%)
Cardiomyopathies	0(0%)	0(0)	14(3.62%)	3(3.13%)

Abbreviations: BSA: body surface area; HR: heart rate; R.HD: rheumatic heart disease.

**TABLE 2:** **Baseline** characteristics of the retrospective database.

	No MR(*N *= 241)		MR(*N *= 492)	
	Training/	Test	Training/	Test
	validation(*n *= 200)	(*N *= 50)	Validation(*N *= 387)	(*N *= 96)
Mild(*n*)	/	/	202(52.20%)	50(52.08%)
Moderate(*n*)	/	/	84(21.70%)	21(21.88%)
Severe(*n*)	/	/	101(26.10%)	25(26.04%)
Central regurgitation(*n*)	/	/	272(70.28%)	62(64.58%)
Eccentric regurgitation(*n*)	/	/	115(29.72%)	34(35.42%)
Echocardiography				
LVEF(%)	65.40 ± 4.46	66.64 ± 3.83	64(58‐68)	65(59‐68)
LVDd(mm)	47.64 ± 4.91	46.58 ± 5.33	52(47‐57)	52.85 ± 9.29
LVSd(mm)	31.73 ± 4.05	30.16 ± 3.91	34(30‐39)	35.30 ± 9.29
IVSd(mm)	9.32 ± 1.34	9.05 ± 1.21	9.3(8.6‐10.3)	9.60 ± 1.52
LAD(mm)	34.60 ± 4.13	33.18 ± 4.11	39(34‐45)	39.66 ± 8.86
E(m/s)	0.87 ± 0.22	0.82 ± 0.21	0.98(0.76‐1.26)	1.02 ± 0.39
A(m/s)	0.79 ± 0.19	0.78 ± 0.17	0.8(0.66‐0.98)	0.81 ± 0.32
Septal e’(cm/s)	8.84 ± 2.20	8.31 ± 2.23	7.27 ± 2.43	7.56 ± 2.46
Latal e’ (cm/s)	11.54 ± 2.81	11.59 ± 3.54	9.93 ± 3.22	10.19 ± 3.44

Abbreviations: LVEF: left ventricular ejection fraction; LVDd: left ventricular end‐diastolic dimension; LVSd: left ventricular end‐systole dimension; LAD: left atrium dimension; IVSd: LV end‐diastolic septal thickness dimension.

**TABLE 3:** **Clinical** characteristics of the prospective test database.

	NO MR(*N *= 70)	MR(*N *= 147)
Age(*y*)	44.81 ± 11.44	53.66 ± 15.00
Male(*n*)	35(50%)	51(34.69%)
Height(cm)	163.14 ± 8.36	160(55‐167)
Weight(kg)	61.41 ± 9.03	56.00(50.00‐65.50)
BSA(*m* ^2^)	1.65 ± 0.16	1.59 ± 0.17
HR(bpm)	76.04 ± 8.25	80.72 ± 17.93
Comorbidities		
Hypertension	23(32.86%)	35(23.80%)
Diabetes	21(30.00%)	28(19.05%)
Atrial fibrillation	0(0)	9(6.12%)
Heart failure	0(0)	11(7.48%)
Rheumatic heart disease	1(1.43%)	11(7.58%)
Cardiomyopathies	1(1.43%)	4(2.72%)

Abbreviations: BSA: body surface area; HR: heart rate.

**TABLE 4:** **Baseline** characteristics of the prospective test database.

	No MR(*N *= 70)	MR(*N *= 147)
Mild(*n*)	/	74(50.34%)
Moderate(*n*)	/	37(25.17%)
Severe(*n*)	/	36(24.49%)
Central regurgitation(*n*)	/	106(72.11%)
Eccentric regurgitation(*n*)	/	41(27.89%)
Echocardiography		
LVEF (%)	65.81 ± 3.74	64(57‐69%)
LVDd(mm)	46.13 ± 3.50	49(45‐54%)
LVSd(mm)	30.07 ± 3.21	31(29‐37)
IVSd(mm)	9.25 ± 1.13	9(8‐10)
LAD(mm)	33.31 ± 3.01	38.76 ± 7.20
E(m/s)	0.86 ± 0.18	0.98(0.78‐1.22)
A(m/s)	0.73 ± 0.19	0.83 ± 0.30
Septal e’(m/s)	8.75 ± 1.95	7.24 ± 2.56
Latal e’ (m/s)	11.84 ± 2.23	9.74 ± 3.47

Abbreviations: LVEF: left ventricular ejection fraction; LVDd: left ventricular end‐diastolic dimension; LVSd: left ventricular end‐systole dimension; LAD: left atrium dimension; IVSd: LV end‐diastolic septal thickness dimension.

### 3.2 **Performance evaluation of** DL **models for detecting MR**

#### 3.2.1 Performance of the AI model in detecting MR in multi‐view

**FIGURE 2:**
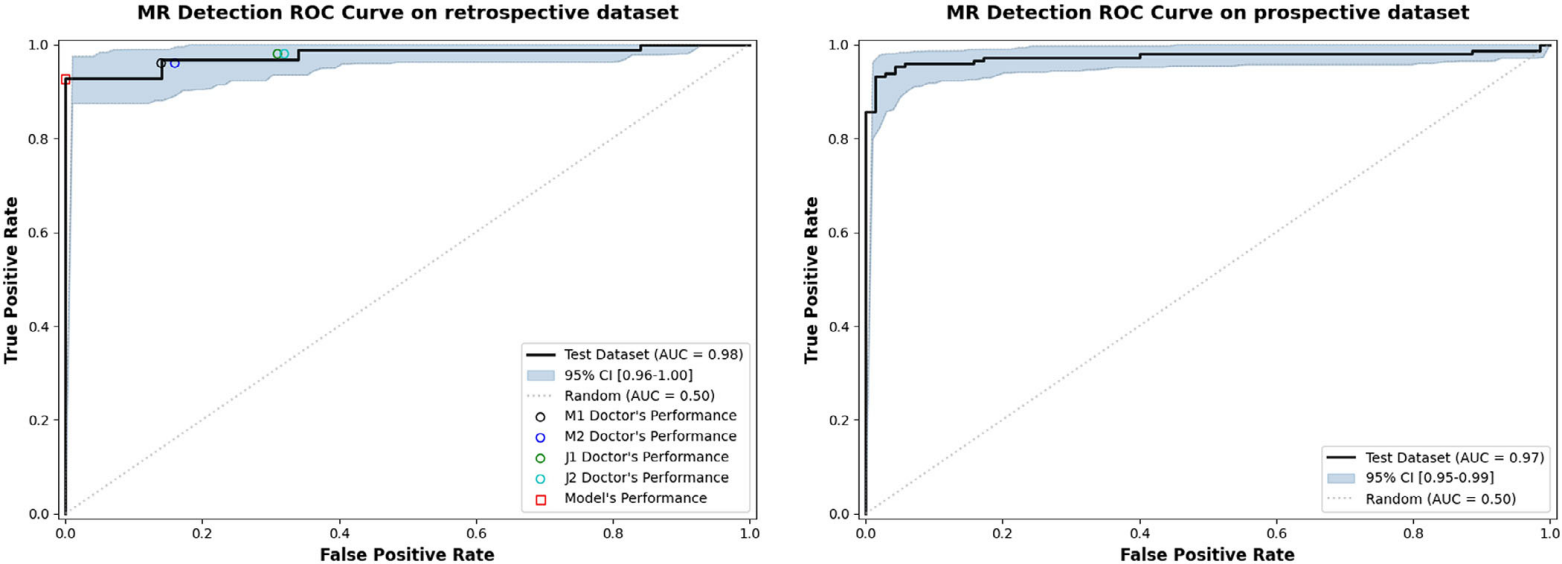
ROC analysis of retrospective and prospective test sets in diagnosing MR. The closed lines represent the performance of the deep learning model on retrospective and prospective data sets respectively. Circles of different colors represent the performance of doctors with different seniority in diagnosing MR. The squares represent the levels of true positive rate and false positive rate when the regurgitation probability threshold is 0.5.

As shown in Table 5, the Transformer achieved high performance in detecting MR. In the retrospective test set, the accuracy reached 0.94 (95%CI:0.90–0.97), and the precision, recall, and F1‐score were 0.98 (95%CI:0.95–1.0), 0.93 (95%CI:0.88–0.98), and 0.95 (95%CI:0.92–0.98), respectively. According to the prospective test set, the MR detection accuracy was 0.92 (95%CI:0.89–0.96), and 0.99 (95%CI:0.97–1.0),0.89 (95%CI:0.83–0.94), and 0.94 (95%CI:0.91–0.97) respectively. The model's performance across the retrospective and prospective test sets is detailed in the confusion matrices (Figure S5). In retrospective test set, our model was compared with doctors of different seniority who had different years of professional work experience. Sensitivity and specificity were calculated as a function of the algorithm's prediction ratio, and the ROC curve and AUC were obtained to quantify the performance of the algorithm Figure 2 and Table S5. The AUC of the retrospectively tested centralized model reached 0.98, and its performance reached that of middle‐aged individuals. Its sensitivity was 0.96, its specificity was 0.90, and its AUC was 0.97 according to the prospective test database. The video‐level performance is reported in Tables S6. Notably, video‐level metrics were slightly lower than patient‐level results, since the multi‐instance learning framework assigns greater weight to patient‐level classification, and the aggregation of multiple video predictions per patient inherently reduces individual video‐level noise.

**TABLE 5:** **Performance** of model for identifying the presence of MR in the retrospective and prospective test database.

Retrospective test database				
Class	Accuracy	Precision	Recall	F1‐score
	(95%CI)	(95%CI)	(95%CI)	(95%CI)
MR(*n *= 96)	0.94(0.90–0.97)	0.98(0.95–1.0)	0.93(0.88–0.98)	0.95(0.92–0.98)
NO MR(*n *= 50)		0.88(0.80–0.95)	0.96(0.90–1.0)	0.92(0.86–0.96)
Prospective test database				
MR(*n *= 147)	0.92(0.89–0.96)	0.99(0.97‐1.0)	0.89(0.83–0.94)	0.94(0.91–0.97)
NO MR(*n * = 70)		0.82(0.73‐0.90)	0.98(0.94–1.0)	0.89(0.84–0.94)

#### 3.2.2 Performance of different models in identifying MR in Retrospective test database

To ensure fairness, we used the same configurations for preprocessing, input length, data augmentation, and pretraining for both the CNN and Transformer models. According to the quantitative comparison results provided in Table S7, Transformer‐based methods significantly outperformed convolutional neural network (CNN)‐based methods in identifying MR on a retrospective test database. Among these models, Swin‐T+DS‐MIL achieved the best performance with an F1 score of 0.92, substantially surpassing both ResNet18+DS‐MIL and ViT‐B+DS‐MIL. When further integrated with the proposed SSA module, Swin‐T+DS‐MIL+SSA attained the best overall results, achieving an accuracy of 0.95, a precision of 1.00, a recall of 0.93, and an F1 score of 0.96. These results clearly demonstrate the effectiveness of both the DS‐MIL and SSA modules in enhancing model performance.

#### 3.2.3 Comparison with large‐scale state‐of‐the‐art models (EchoNet/MR[12])

To further validate our method, we evaluated the open‐source EchoNet/MR model using an internal dataset. Although EchoNet/MR's training dataset is larger (*n* = 58 614), it primarily relies on single‐view (A4C‐Color) videos. As shown in Table 6, our model, trained with only 513 samples, achieved a competitive AUC and a higher F1‐score in retrospective evaluation (confusion matrix referenced in Figure S14). Notably, our multi‐view model exhibits more balanced performance compared to EchoNet/MR (F1‐score: 0.93–0.94). This demonstrates that incorporating multi‐view information can capture more comprehensive spatial features for MR evaluation.

**TABLE 6:** MR detection performance was compared with state‐of‐the‐art EchoNet/MR models on both retrospective and prospective databases.

Retrospective test database						
Methods	Training samples	Views	AUC	Precision	Recall	F1‐score
Echonet/MR	58 614	A4C‐Color	0.97(0.91–1.0)	0.82(0.65–0.96)	0.95(0.82–1.0)	0.88(0.76–0.97)
Ours	513	A4C‐Color	0.96(0.87–1.0)	0.94(0.83–1.0)	0.91(0.78–1.0)	0.92(0.83–0.98)
Ours	513	Multi‐view	0.97(0.90–1.0)	0.96(0.86–1.0)	0.91(0.78–1.0)	0.93(0.85–1.0)
Prospective test database						
Echonet/MR	58 614	A4C‐Color	0.98(0.95–0.99)	0.98(0.93–1.0)	0.93(0.85–1.0)	0.95(0.90–0.99)
Ours	513	A4C‐Color	0.97(0.92–0.99)	0.98(0.94–1.0)	0.88(0.79–0.95)	0.93(0.87–0.97)
Ours	513	Multi‐view	0.98(0.94–0.99)	0.99(0.96–1.0)	0.89(0.80–0.96)	0.94(0.88–0.98)

#### 3.2.4 Advantages of DL models in multi‐view detection of MR

For fair comparison, patient samples with PLAX‐Color and A4C‐Color views were selected from the original database to form a sub‐database to verify the effectiveness of multi‐view diagnosis, with a training set (*n* = 325) and a test set (*n* = 92). As shown in Table 7, in the retrospective data set, the accuracy of the model in detecting MR in the single PLAX‐Color and A4C‐Color was approximately 0.82 and 0.91, respectively. The accuracy of multi‐view comprehensive detection of MR can reach 0.92, and it also has advantages in the F1‐score of the regurgitation category (Figure S6). This superiority of MR multi‐view diagnosis persisted in the prospective database with an accuracy of 0.92, compared with approximately 0.89, and 0.92 for PLAX‐Color and A4C‐Color, respectively (Figure S7).

**TABLE 7:** Performance of model for detecting MR in the plax‐color, a4c‐color, and multi‐view for retrospective and prospective test database.

Retrospective test database				
View (*n *= 65)	Accuracy	Precision	Recall	F1‐score
PLAX‐color	0.82	0.85	0.89	0.87
A4C‐color	0.91	0.94	0.94	0.94
Multi‐view	0.92	0.94	0.95	0.95

Prospective test database				
View (*n *= 131)	Accuracy	Precision	Recall	F1‐score
PLAX‐color	0.89	0.91	0.95	0.93
A4C‐color	0.92	0.94	0.95	0.94
Multi‐view	0.92	0.95	0.95	0.95

#### 3.2.5 Class activation mapping technology is also used to understand the learning process of the visual MR detection model

**FIGURE 3:**
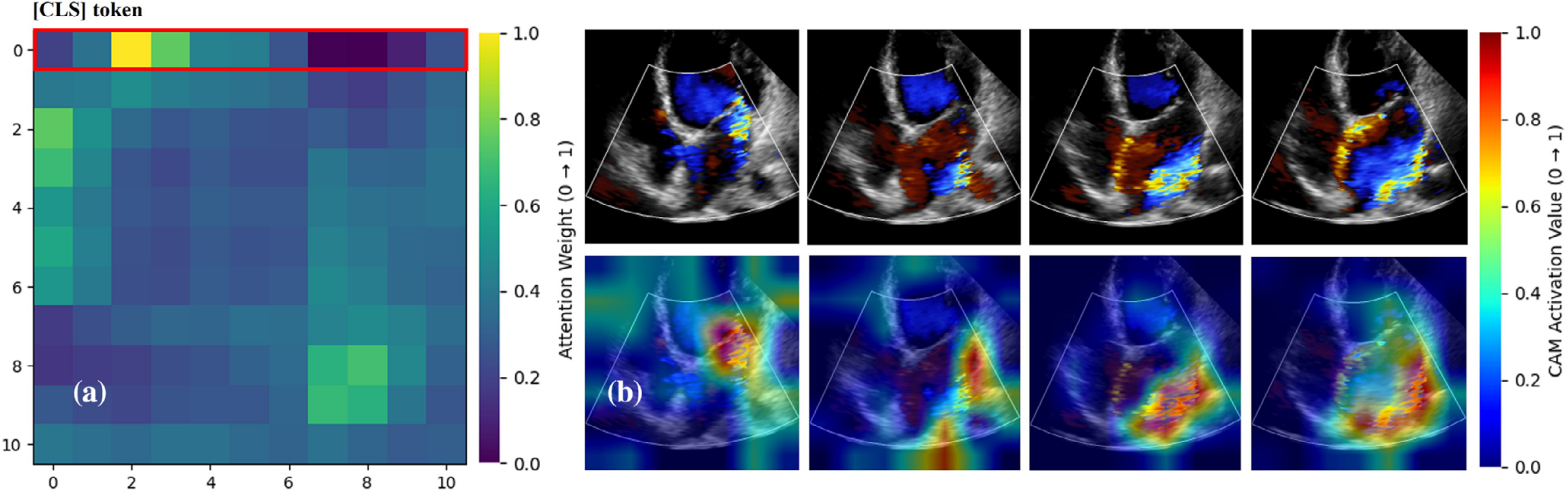
Activation maps of the attention‐based MR Detection model in Case 1 (A4C‐Color). (a) Self‐attention matrix covering 11 input frames, where index 0 corresponds to the [CLS] token. MR events occur between frames 1–6; row 0, highlighted by the red box, shows the attention weight assigned to each frame by the [CLS] token—warmer colors indicate higher attention (see color bar on the right). The model exhibits high attention to frames 1–6, consistent with clinically relevant MR periods. (b) Top row: Raw color Doppler echocardiographic frames from the MR sequence. Bottom row: Corresponding attention heatmaps superimposed on the same frames. The heatmaps show that the model primarily attends to the color Doppler information from the mitral valve orifice to the left atrium.

**FIGURE 4:**
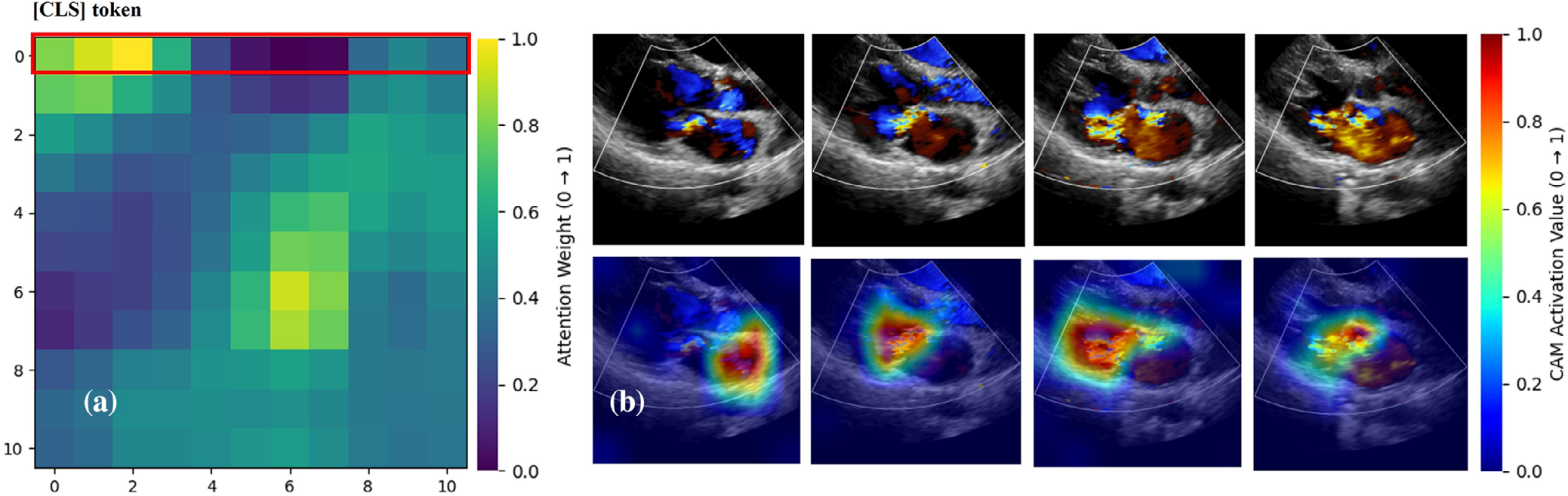
Activation maps of the attention‐based MR Detection model in Case 2 (PLAX‐Color). (a) Self‐attention matrix covering 11 input frames, where index 0 corresponds to the [CLS] token. MR events occur between frames 1–5; row 0, highlighted by the red box, shows the attention weight assigned to each frame by the [CLS] token—warmer colors indicate higher attention (see color bar on the right). The model shows high attention to frames 1–4, which happen to be MR frames. (b) Top row: Raw color Doppler echocardiographic frames from the MR sequence. Bottom row: Corresponding attention heatmaps superimposed on the same frames. The heatmaps show that the model can pay attention to the color Doppler information corresponding to the position of the mitral valve.

As shown in Figures 3 and 4, through spatiotemporal heatmaps, combining the attention matrix across 11 frames (with frame 0 as the [CLS]token^18^ and pixel‐level activations within each frame, we observe that the model selectively attends to frames containing MR and concentrates its activation on the regurgitation jet region. This indicates that the model's predictions are grounded in clinically relevant patterns: namely, the spatial path of the color Doppler signal near the mitral valve orifice, and its temporal persistence across MR frames. By revealing where in space and when in time the model focuses, these visualizations provide transparent, human‐interpretable insight into its decision logic, thereby enhancing model interpretability.

### 3.3 **Performance of the** DL **model for centrality or eccentricity MR classification**

#### 3.3.1 DL performance in classifying central or eccentric MR

**FIGURE 5:**
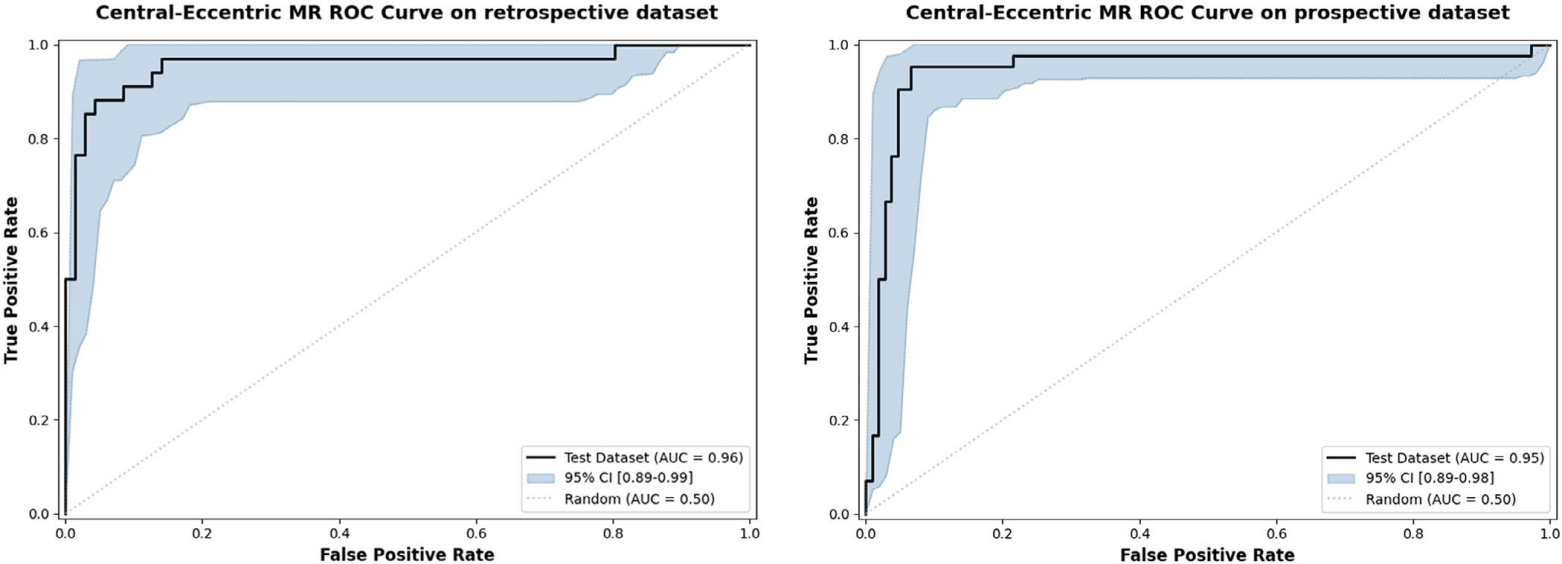
ROC analysis of retrospective and prospective test sets in diagnosing central‐eccentric MR.

As shown in Table 8, the transformer also achieved high performance in classifying central and eccentric MR. The accuracy was 0.93 in the retrospective and prospective test sets. The ROC curve and AUC were used to quantify the performance of the algorithm, and the AUC was 0.96 (95%CI:0.89–0.99) and 0.95 (95%CI:0.89–0.98) in the retrospective and prospective test sets (Figure 5). Figure S8 illustrates the confusion matrices, detailing the model's classification performance on both the retrospective and prospective test sets.Similarly, the video‐level classification performance is reported in Table S8, with a consistent trend as observed in MR detection.

**TABLE 8:** **Performance** of model for classifying central or eccentric MR in the retrospective and prospective test database.

Retrospective test database				
Class	Accuracy (95%CI)	Precision (95%CI)	Recall (95%CI)	F1‐score (95%CI)
Central MR(*n *= 62)	0.93(0.87–0.97)	0.93 (0.86–0.98)	0.97 (0.92–1.0)	0.95 (0.90–0.98)
Eccentric MR(*n *= 34)		0.930(0.81–1.0)	0.85 (0.72–0.95)	0.89 (0.78–0.95)
Prospective test database				
Central MR(*n *= 106)	0.93 (0.89–0.97)	0.96 (0.91–0.99)	0.95 (0.91–0.99)	0.95 (0.92–0.98)
Eccentric MR(*n *= 41)		0.88 (0.76–0.97)	0.89 (0.77–0.97)	0.88 (0.78–0.94)

#### 3.3.2 Advantages of DL models in multi‐view classification of central or eccentric MR (Table 9)

Similar to the detection of MR, patient samples with both PLAX‐Color and A4C‐Color views were selected from the original data set to form sub‐datasets (training set *n* = 239, test set *n* = 71). In the retrospective data set, the model's classification accuracy in left ventricular single long‐axis view, apical four‐chamber view (A4C), and multi‐view diagnosis were approximately 0.86, 0.86, and 0.90 respectively, while the F1‐score of the eccentric MR category was 0.78, 0.81, and 0.87 respectively (Figure S9). The classification accuracy of the prospective database was 0.90, 0.91, and 0.91 respectively, and the F1‐score of the eccentric MR category was 0.82, 0.84, and 0.86 respectively (Figure S10). Due to data imbalance, the F1‐score of the eccentric MR can better highlight the advantages of multi‐view diagnosis.

**TABLE 9:** **Performance** of model for classifying central or eccentric MR in the PLAX‐Color, A4C‐color, and multi‐view for retrospective and prospective test database.

Retrospective test database					
	View (*n *= 71)	Accuracy	Precision	Recall	F1‐score
PLAX‐color	Central MR(*n *= 45)	0.86	0.84	0.96	0.90
	Eccentric 0MR(*n *= 26)		0.90	0.69	0.78
A4C‐color	Central MR(*n *= 45)	0.86	0.89	0.89	0.89
	Eccentric MR(*n *= 26)		0.81	0.81	0.81
Multi‐view	Central MR(*n *= 45)	0.90	0.93	0.91	0.92
	Eccentric MR(*n *= 26)		0.85	0.88	0.87

Prospective test database					
	View (*n *= 131)	Accuracy	Precision	Recall	F1‐score
PLAX‐color	Central MR(*n *= 93)	0.90	0.92	0.95	0.93
	Eccentric MR(*n *= 38)		0.86	0.79	0.82
A4C‐color	Central MR(*n *= 93)	0.91	0.93	0.95	0.94
	Eccentric MR(*n *= 38)		0.86	0.82	0.84
Multi ‐view	Central MR(*n *= 93)	0.91	0.98	0.89	0.93
	Eccentric MR(*n *= 38)		0.78	0.95	0.86

#### 3.3.3 Class activation mapping technology has also been used to predict central or eccentric MR classification models

**FIGURE 6:**
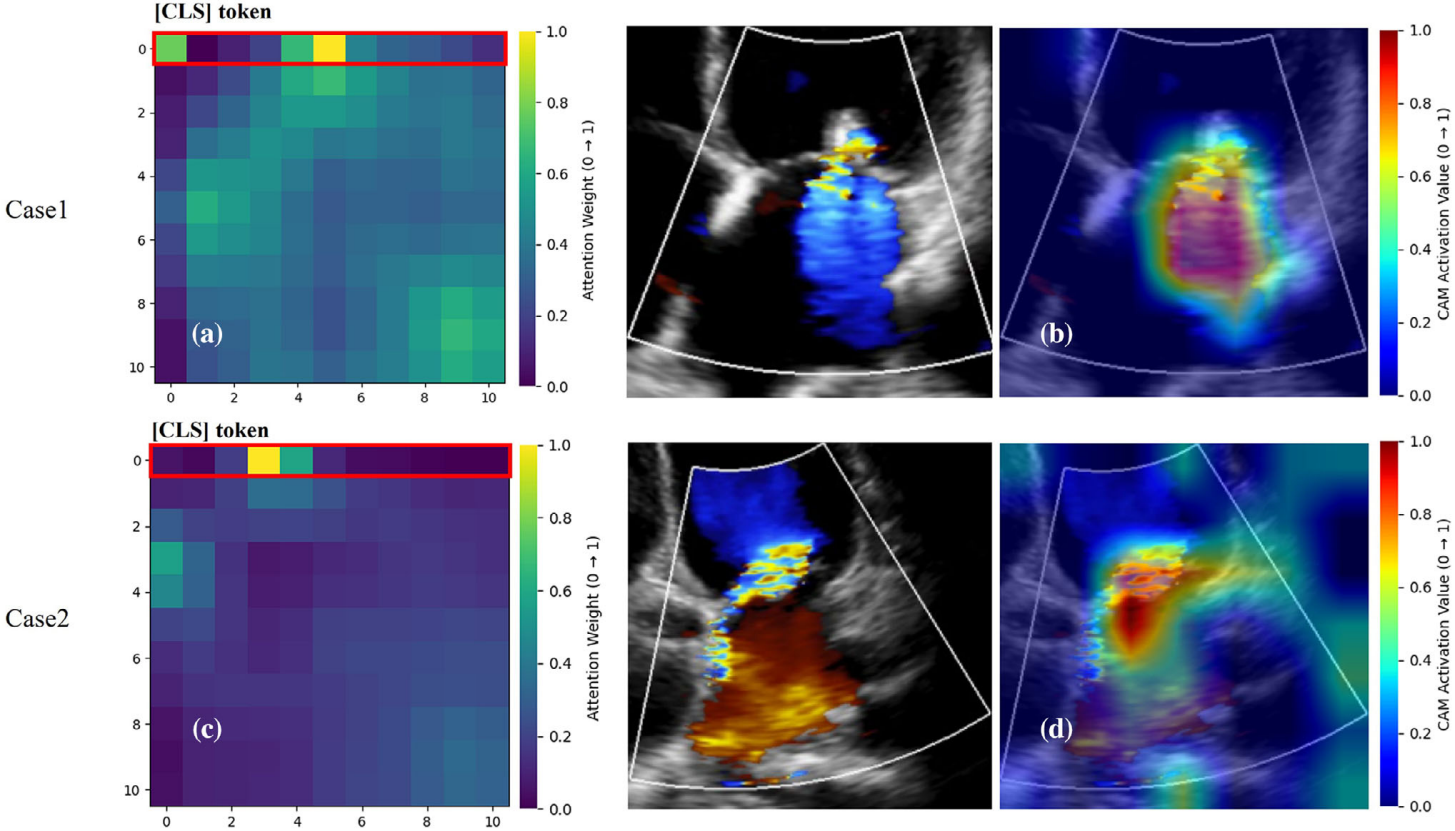
Activation maps of the attention‐based central‐eccentric MR classification model (Case1‐central MR and Case2‐Eccentric MR). (a) and (c) Attention matrices across 11 input frames (frame 0 is [CLS] token) for two representative cases: The highlighted 0th row (top row, red box) indicates the attention weights assigned by the [CLS] token to each frame. Warmer colors (yellow—red) denote higher attention scores (see color bar on the right), while cooler tones (blue—green) indicate lower attention. In both cases, the model predominantly attends to the frame with maximal regurgitant jet intensity—consistent with clinically relevant timepoints. (b) and (d) Left: Original color Doppler echocardiographic images of the maximum regurgitation frame. Right: Corresponding attention heatmaps overlaid on the same frames show that the model can pay attention to the shape of MR in the left atrial surface.

As shown in Figure 6, that the model's attention primarily centers on the regurgitation frames, with spatial activations highlighting the MR shape of the left atrial surface, precisely where clinicians visually assess jet directionality. This demonstrates that the model does not rely on arbitrary patterns, but learns to differentiate central from eccentric MR based on anatomically and dynamically meaningful features. The visualizations expose what drives classification, increasing explainability and clinical acceptance.

### 3.4 Explainable analysis of occlusion and ablation in color doppler regions

To verify the model's reliance on the color Doppler region and the interpretability of its decisions, we performed systematic regional ablation experiments. As illustrated in Figure S11, we incrementally applied random masking to the color Doppler region using 16×16 pixel blocks, with the mask ratio progressively increased from 0 to 30%. Masking was prioritized in the central image area, and model performance was evaluated throughout.

Figure S12 shows a progressive decline in classification performance with increasing masking. MR detection accuracy dropped from 0.94 to 0.69 and AUC from 0.98 to 0.81, with expanding 95% CIs, and central‐eccentric MR showed the same declining pattern. This indicates rising prediction uncertainty with the loss of relevant regional information. These findings confirm that the model depends substantially on hemodynamic features from the color Doppler region for its decisions. The results align with attention map visualizations, further supporting the interpretability and reliability of the model's decision‐making process.

### 3.5 Clinical workflow integration and inference efficiency

Our model is designed for seamless integration into standard echocardiography workflows. It operates at the stage between image acquisition and formal diagnosis, functioning as an automated pre‐reading triage tool. Following the clinical diagnostic pathway, it first detects the presence of MR, then classifies positive cases into central or eccentric types, aiding rapid case prioritization and feature highlighting.

On a single NVIDIA A6000 GPU, the model achieves an end to end inference time of 1.05 seconds (95%CI:0.92–1.18s) per study, which includes processing multiple video clips (Figure S13). This efficiency supports near real time analysis, making it feasible for integration into routine clinical or point of care workflows without disrupting diagnostic throughput.

## 4 Discussion

**FIGURE 7:**
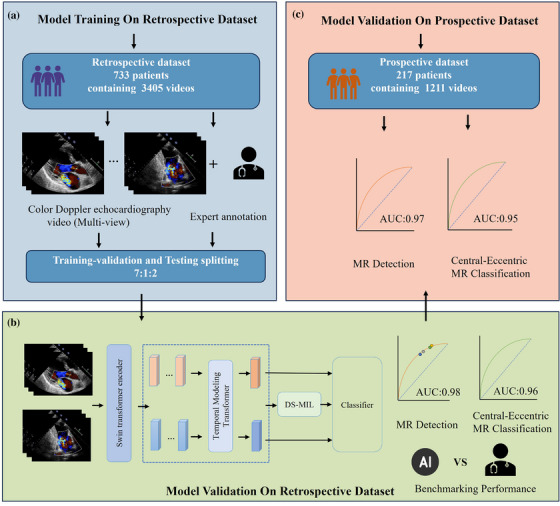
**Central Illustration** (a) Using a single‐center retrospective database, we built two deep learning models based on Swin Transformer multi‐view diagnosis to detect MR and identify central regurgitation or eccentric regurgitation respectively; (b) Deep learning model workflow and internal validation in a retrospective test set. In addition, we compared the performance of the model with the diagnoses of physicians with different seniority; (c) Our model was also validated in a prospective test set.

This study introduced a Transformer‐based algorithm capable of fully automatically analyze multi‐view color Doppler videos for screening the presence of MR and determining the regurgitation characteristics of lesions when regurgitation is present. The performance of the DL model developed using a retrospective database for detecting MR and classifying MR as eccentric or central regurgitation was validated in a prospective dataset of actual consecutive patients. This algorithm achieved an accuracy comparable to some experienced doctors in detecting the presence of mitral valve disease and classifying regurgitation characteristics, and its performance is better than that of a single view. Although physiological MR is not always clinically significant, our model demonstrated that it is feasible to use AI to screen for MR in adults and classify regurgitation characteristics (Figure 7).

### 4.1 Application of the transformer in detecting MR

There are problems of subjective bias and high cost in echocardiographic analysis. DL algorithms have been developed to help human experts complete these tasks. Most of those previous works were based on the CNN structure and obtained good results. In recent years, visual Transformation models have demonstrated their effectiveness and generalizability in traditional semantic segmentation tasks.^19^, ^20^ In image segmentation, studies have shown that^21^, ^22^, ^23^ introducing the Transformer into computer vision can rival or even exceed the performance of CNNs, thus opening up new avenues for the development of computer vision. However, the Swin Transformer has not been used to explore MR in existing studies. Our study shown that the Swin Transformer achieves high performance in detecting MR from Doppler videos, with accuracy matching considerable experienced clinicians and validated the superior performance of our proposed Transformer‐based network over the traditional CNN architecture. Therefore, the algorithm's risk of misclassifying the presence or absence of MR is comparable to that encountered in current clinical practice, and the algorithm may perform better than less experienced physicians.

The use of DL in health care is complicated by uninterpretable black box models. Artificial neural networks are often uninterpretable because the relationships among their multiple layers of interconnected variables are nonlinear. If the reasoning behind medical decisions is unclear, clinicians and patients may be reluctant to blindly trust the conclusions.^24^ Explainability is a basic attribute of AI systems and the basis for achieving transparency, accountability, and other ethical values. As demonstrated in Figures 3 and 4, our model showed spatiotemporal attention heatmaps for the MR detection task, revealing that it successfully identifies frames containing MR within each video segment and focuses its activation on the most clinically relevant features. Our results shown that DL model mainly focuses on the brightest activation signals in Doppler videos, which are the color Doppler signals of MR and the regurgitation trajectory in left atrial. These signals display high contrast and distinct borders in the Doppler spectrum, crucial for classification tasks. The model's emphasis on these key features for assessing MR and eccentricity reflects clinical priorities. It effectively leverages these signals to capture diagnostic features that are consistent with established clinical practices. Moreover, our model can focus on the number of frames with MR information in a cardiac cycle and assist in determining the location of key frames, laying an important preliminary foundation for subsequent automatic judgment of the degree of MR information and for semiquantitative and quantitative analysis.

### 4.2 The advantages of comprehensively detecting MR from multiple views

AI can not only provide support for sonographers to obtain standard views,^25^, ^26^ but also assist in identifying potential defects in the mitral valve and evaluating valve function. Due to the variability, similarity, complexity and other factors within and between views, previous studies have used only static US images or a single frame rate or view to screen adult MR with high accuracy.^8^, ^9^, ^10^ Another study used a CNN model to identify the presence of MR in the long‐axis view of the left ventricle of children using color Doppler, with an accuracy of 0.86.^27^ However, in diagnosing and evaluating MR, it is necessary to obtain comprehensive judgments from multiple views. In our study, the multi‐view fusion model established by using a relatively complete video as input data is very useful for diagnosing MR. Our model achieved an accuracy of 0.94 in both the retrospective test database and the prospective test set, with had high sensitivity and specificity. To make a fair comparison, we selected cases with both PLAX‐Color and A4C‐Color from the original dataset to constitute a sub‐dataset for training and testing, which showed that regardless of whether multiple views were used to diagnose MR in retrospective or prospective datasets, the accuracy of the classification or F1‐score of the target class was greater than those using only a single view. As with MR detection, the accuracy or F1‐score performance of multi‐view diagnosis in identifying MR characteristics in both retrospective and prospective datasets was greater than that of diagnosing only a single view. Despite differences in image quality due to differences in echocardiography windows between subjects and technical differences between scanners, our study demonstrated high accuracy, and rather than focusing on a single anatomical structure, our method allows for a broader classification that is more consistent with the clinical practice process of diagnosing MR.

Combining the multisource information of these multiple dynamic videos to form a more comprehensive and rich feature representation can largely solve the problems of multi‐view, difficulty in analysis, and low stability and repeatability with the cardiac cycle in daily work, greatly improving sonographers’ work efficiency and diagnostic accuracy. The development of AI has improved US diagnosis accuracy, reduced misdiagnosis rates, shortened reporting times, and met growing clinical needs. Therefore, this model has the potential to serve as a screening tool to help healthcare professionals identify patients with MR, particularly in areas with limited access to expert physicians.

### 4.3 Analysis of the advancement of central or eccentric MR classification

Depending on the mechanism and severity of the disease, MR can manifest as central or eccentric regurgitation. The direction of MR in the left atrium can attract the attention of sonographers to a certain extent, thereby helping to diagnose the pathogenesis of MR, such as acute papillary muscle rupture, chordae tendineae rupture, and valvular vegetation, which often necessitate further emphasis on the urgency and method of surgical intervention. Eccentric MR can reflect the course and prognostic value of functional MR to a certain extent.^28^ The 2020 ACC/AHA and 2021 ESC valve management guidelines both indicate that in chronic MR, eccentric and central regurgitation have different reference meanings.^29^, ^30^ However, eccentric regurgitation has unique hemodynamics, and it is difficult to observe the entire jet with color Doppler. Quantitative and semiquantitative methods that can usually be used to evaluate central regurgitation often underestimate the degree of regurgitation when evaluating eccentric regurgitation.^31^, ^32^, ^33^, ^34^ So we need to focus on whether the mitral valve is eccentric regurgitation or central regurgitation. An economical, convenient and accurate method to evaluate mitral eccentric regurgitation is needed in the clinic.

To the best of our knowledge, this study is the first to use an AI model to identify central or eccentric MR in multi‐view echocardiography videos. The accuracy was 0.93 in both the retrospective and prospective datasets. This study introduced a Transformer‐based eccentricity classification framework for MR that automatically analyzes multi‐view echocardiographic Doppler videos and identifies eccentric regurgitation through integrated multi‐view representation. Specifically, the model allows multiple view videos to be input in any combination or order and can flexibly handle the common problem of missing views in clinical applications. It also uses dual‐layer supervision at the video and case levels to improve the classification performance of the model effectively. The goal of fully automated clinical screening should be achieved. Model decision‐making interpretability can be enhanced by showcasing the attention matrices in temporal and spatial domains throughout the model prediction process. This visualization approach spotlights the features the model prioritizes at various time steps and spatial locations, bolstering the transparency and trustworthiness of the decision‐making. Our research demonstrated that AI can identify MR and determine its characteristics, offering a valuable auxiliary tool for screening. This approach can be used as an auxiliary tool for screening and simplifying workflow and management, thereby improving work efficiency. The reasonable allocation of limited resources can, in particular, reduce diagnostic errors caused by relying on the subjective experience of sonographers. In addition, these findings provide an important preliminary basis for subsequent quantitative analysis of central or eccentric MR.

**Limitations**: Our study has several limitations: First, during model training, we mainly used high‐quality images to optimize learning efficiency and improve classification accuracy. However, inferior‐quality or incomplete images, common in clinical practice, can negatively impact the model's performance and doctors' diagnostic decisions. In addition, we have noticed that domain discrepancy issues caused by different vendors or probes may affect the model's performance on cross‐domain data. Our current test set only contains data from a single hospital, and cross‐vendor variability across different hospitals is a key factor affecting the model's generalization ability. Therefore, in our future work, we plan to introduce domain‐invariant learning methods^35^ to improve the model's generalization ability on unseen domains by learning shared, domain‐independent feature representations across different domains. Second, the sample size was relatively small, and there were phenomena such as missing data in the retrospective dataset. Our test set currently includes data from only one hospital, and there is no additional external verification to evaluate the performance of the model. Third, our model currently determines only whether there is MR and central or eccentric regurgitation and does not further divide it into mild, moderate, severe, semiquantitative, or quantitative parameter assessments. However, we are already in the abovementioned research process. In the end, our model initialization used the ImageNet dataset pretraining parameters, and we will study pretraining tasks that are more suitable for medical US images later.

## 5 Conclusion

We successfully established an AI model based on a Transformer to automatically evaluate MR from multiple views and determine central or eccentric regurgitation. The early success of our method shown that automatic MR detection and classification of regurgitation characteristics are feasible, which also demonstrated the potential of Transformer‐based models in cardiac echocardiography‐based disease diagnosis.

## AUTHOR CONTRIBUTIONS

**Dong Ni, Wufeng Xue**: Conceptualization and writing—review. **Yuanyuan Sheng, Ying Guo, Yumei Yang, Yuxiang Huang, Hongjuan Zhao, Wei Zeng, Jie Deng, and Lixin Chen**: Data curation and data annotation. **Jinfeng Xu, Yingying Liu**: Supervision and data annotation review. **Jiancheng Zhang**: Methodology and result analysis. **Xiaofang Zhong**: Resources, data curation, and writing—design.

## CONFLICT OF INTEREST STATEMENT

The authors declare that they have no known competing financial interests or personal relationships that could have appeared to influence the work reported in this paper.

## ETHICAL APPROVAL

This study was approved by the Institutional Review Board of the Shenzhen People's Hospital, specifically the Medical Ethics Committee of Shenzhen People's Hospital. All methods were carried out in accordance with relevant guidelines and regulations.

## Supporting information



Supporting Information

## Data Availability

Data will be made available on request.
