# Correction to “Mitral regurgitation detection and central/eccentric classification using transformer‐based deep learning in multi‐view echocardiography”

**DOI:** 10.1002/acm2.70616

**Published:** 2026-05-14

Zhong X, Zhang J, Sheng Y, et al. Mitral regurgitation detection and central/eccentric classification using transformer‐based deep learning in multi‐view echocardiography. *J Appl Clin Med Phys*. 2026;27:e70584. doi:10.1002/acm2.70584

**Description of error**

The affiliation information of the corresponding authors in the original article is incorrect:

**Correspondence**

Jinfeng Xu and Yingying Liu, Department of Ultrasound, Shenzhen People's Hospital (The Second Clinical Medical College, Jinan University; The First Affiliated Hospital), Shenzhen Medical Ultrasound Engineering Center, Shenzhen, 518020, China. Email: jinfengxu@ext.jnu.edu.cn and yingyingliu@ext.jnu.edu.cn

Wufeng Xue, National‐Regional Key Technology Engineering Laboratory for Medical Ultrasound, School of Biomedical Engineering, Shenzhen University Medical School, Shenzhen University, Shenzhen, China. Medical Ultrasound Image Computing (MUSIC) Laboratory, Shenzhen University, China. Marshall Laboratory of Biomedical Engineering, Shenzhen University, China. Email: xuewf@szu.edu.cn

The corrected affiliation information of the corresponding authors is as follows:

**Correspondence**

Jinfeng Xu and Yingying Liu, Department of Ultrasound, Shenzhen People's Hospital (The First Affiliated Hospital, Southern University of Science and Technology; The Second Clinical Medical College, Jinan University), Shenzhen 518020, China. Email: jinfengxu@ext.jnu.edu.cn and yingyingliu@ext.jnu.edu.cn

Wufeng Xue, The National‐Regional Key Technology Engineering Laboratory for Medical Ultrasound, School of Biomedical Engineering, Health Science Center, Shenzhen University, Shenzhen, China; Medical Ultrasound Image Computing (MUSIC) Laboratory, Shenzhen University, Shenzhen, China; Marshall Laboratory of Biomedical Engineering, Shenzhen University, Shenzhen, China. Email: xuewf@szu.edu.cn

Previously, we had checked and confirmed the authors' affiliation information, but due to an oversight, we failed to timely modify the corresponding author's affiliation, resulting in an error in the corresponding author's affiliation in the published article. We apologize for this error. Here, we formally apply to correct the corresponding author's affiliation. We sincerely apologize for any inconvenience caused to you.

