# Supplementary material for: Mitral regurgitation detection and central/eccentric classification using transformer‐based deep learning in multi‐view echocardiography
Source: J Appl Clin Med Phys. 2026 Apr 20;27(4):e70584. doi: 10.1002/acm2.70584 (PMC13092907; doi:10.1002/acm2.70584)
Supplement: Supplementary file 1 — Supporting Information [file ACM2-27-e70584-s001.docx]

**Supplementary materials**


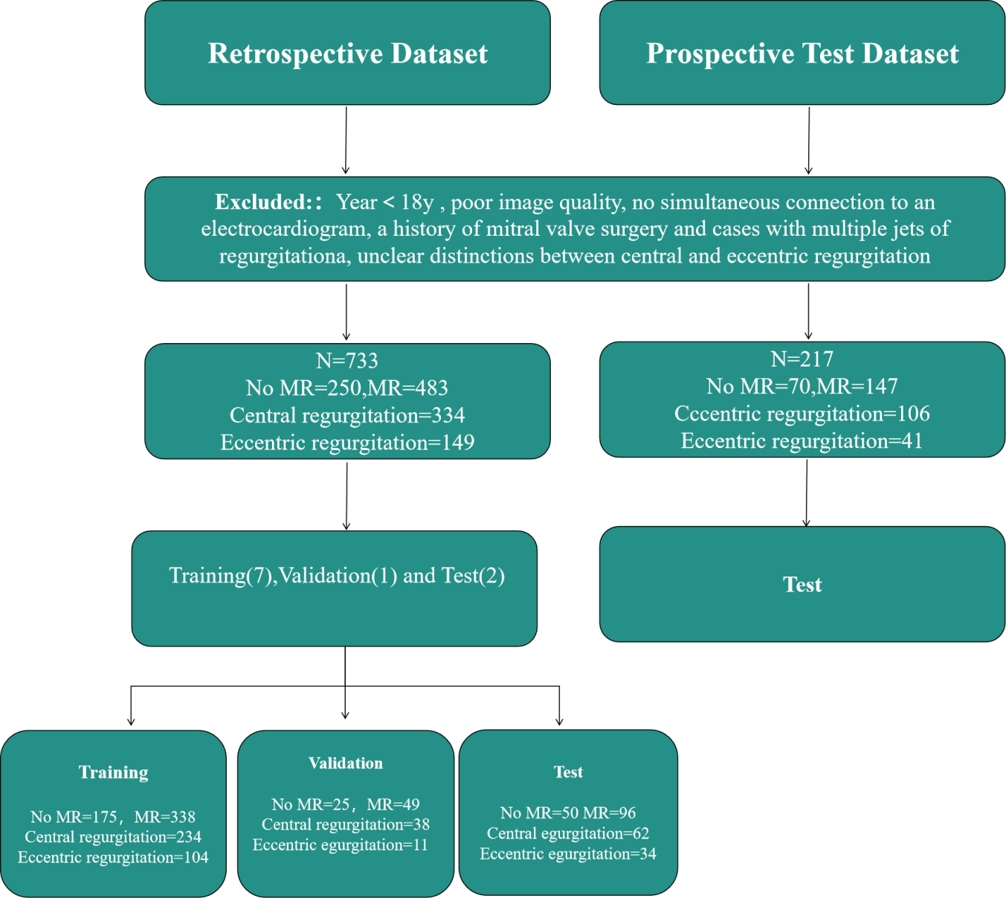


**Supplemental Figure 1:**Summary of Number of Echocardiograms Used in this Study

Criteria for grading the severity of MR

Mitral regurgitation is defined according: to 2017 ASE Recommendations for Noninvasive Evaluation of Native Valvular Regurgitation confirming the presence, severity, and etiology of MR.

Mild: small central jet area ＜20% LA, on Doppler, Small vena contracta <0.3cm

Moderate: Central jet MR 20%-50% LA of late systolic eccentric jet MR; Vena contracta＜0.7cm; Regurgitant volume <60 ml; EROA＜0.40 cm²

Severe: Central jet MR >50% LA or holosystolic eccentric jet MR;Vena contract≥7cm; Regurgitant volume ≥60 ml; EROA≥0.40 cm².

**Swin Transformer Network as Encoder**

As shown in Supplemental Figure 1, Swin Transformer divides color echocardiograms into non-overlapping 4×4 pixels patches. The feature dimension of each patch is 4×4×3=48 and is regarded as a “token”. In stage 1 of the encoder, these patches with raw features are projected into arbitrary dimensions (C) through a linear embedding layer. The arbitrary dimension (C) represents the information capacity embedded in each token. Using the Swin-Tiny configuration, C is set to 96. Each pair of Swin Transformer blocks calculates the self-attention between the patch and shift windows within the M×M window. The Transformer block maintains the number and dimensions of tokens, and the feature map in stage 1 is H/4×W/4×C. Then, in stage 2, adjacent 2×2 patches are connected by a patch merging layer for hierarchical feature representation. This increases the dimensionality to 4C while employing a linear layer to set the output dimensionality to 2C and reducing the tokens to H/8×W/8. The two Transformer blocks will then compute window attention between these larger tokens. This process consists of patch merging layers and several Swin Transformer blocks, repeated twice as "Phase 3" and "Phase 4". The final feature map output by the Swin Transformer encoder is H/32×W/32×8C.


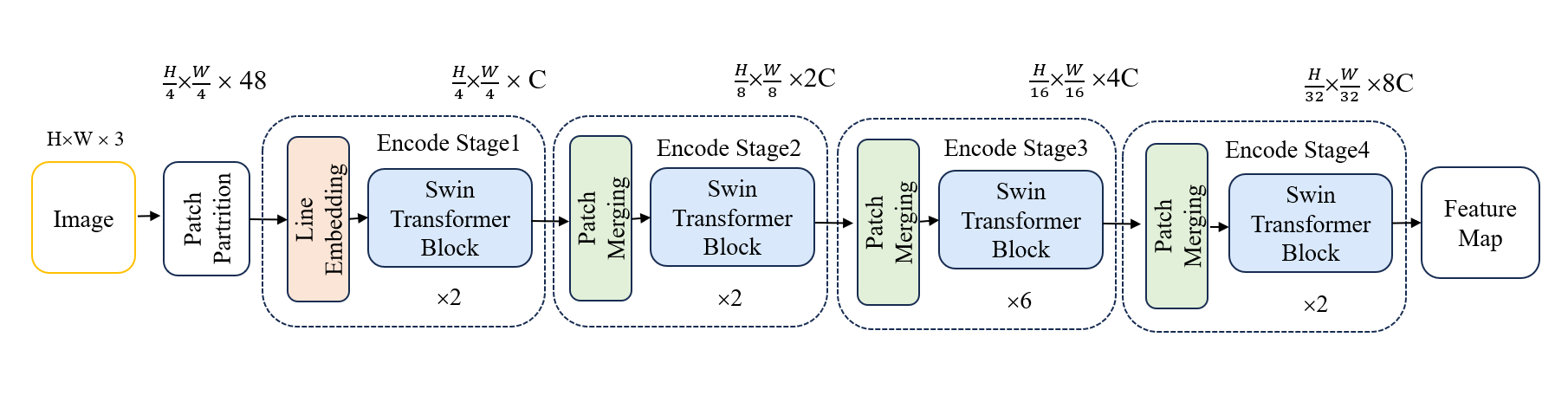


**Supplemental Figure 2** Structure of Swin Transformer backbone network

**Transformer for MR detection and central-eccentric MR classification**

Our system workflow is shown in Figure 1, which consists of two Transformers, a DS-MIL, and a classifier. MR detection and central-eccentric MR classification use the same model architecture. We used Python's Keras (version 2.0.9) library (version 3.8), and Pytorch (version 1.3.0) to build the model. The model is trained with a fixed-size input of 10 multi-view 10-frame video clips (if the sample is less than 10 multi-view videos, we repeat the video until 10), with a resolution of 224 x 224 pixels per frame. First, Swin Transformer Tiny is used as the backbone to extract the features of each frame respectively, and then it is input into the timing modeling module with a 3-layer Transformer block and self-attention head number of 12 to obtain the feature expression of each video clip. The features of multiple video clips are input into the DS-MIL module. As shown in Supplementary Figure 2, any number of video features can be fused through the Max Pool operation (selecting the most expressive features) to obtain patient-level feature expressions, and video-level and patient-level prediction results can be obtained through a shared classifier. Therefore, a dual supervision mechanism of video and patient-level labels can be used for the classifier to improve the classification performance of the model. The total training loss is composed of the cross entropy loss at the patient level and video level, and the self-attention mechanism constraint loss(Supplementary Figure 3), with weights of 0.8, 0.1, and 0.1 respectively. The cosine decay rate scheduler and the linear warmup AdamW optimizer with 5 cycles are used for 60 epochs. The batch size is set to 4. Since the Transformer model needs to be pre-trained on a huge data set to achieve high performance, we use the parameters pre-trained on ImageNet1K to initialize the backbone, and other parts are initialized randomly. Setting the learning rate of the backbone to 2e-5 and the learning rate of other modules to 2e-4 can improve the generalization ability of the model and set the weight decay to 0.01. At the same time, the MR detection and central or eccentric MR classification models are trained and tested independently. For model testing, the model can input any number of view videos, and slide 10 frames for each video for sampling testing. During the test, slide 3 times, and the maximum value of the 3 prediction results is used as the final prediction result.


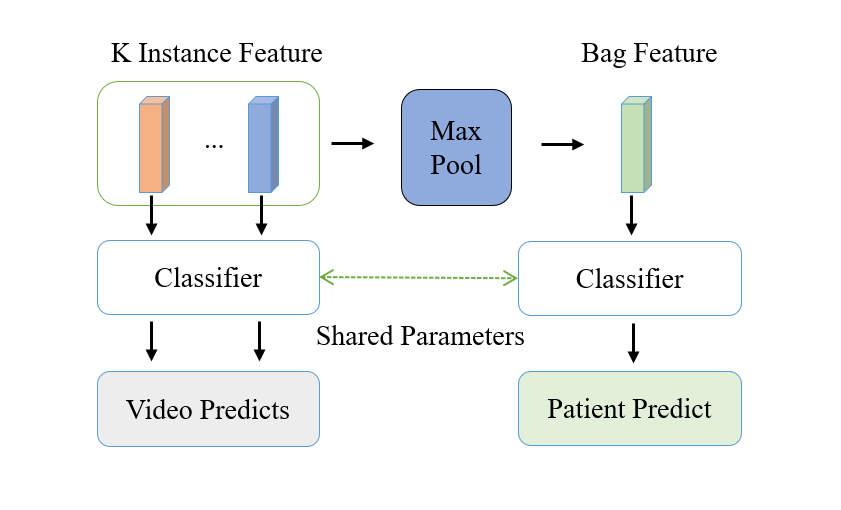


**Supplemental Figure 3** Structure of Dual-level supervised multiple instance learning block


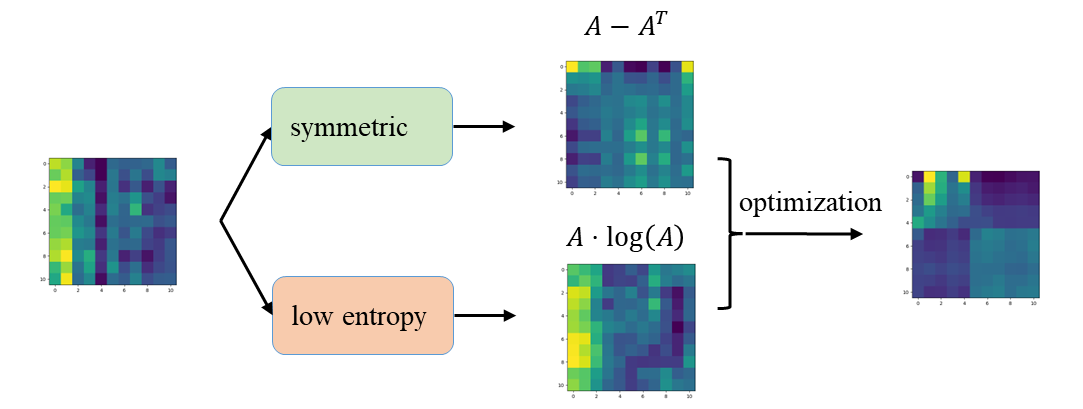


**Supplemental Figure 4** Self-attention matrix constraints (approximate symmetry and low information entropy)

**Interpretation of the Self-Attention Matrix**

In the Transformer layer of temporal modeling, we represent the features of each frame as a token and add the 0th token in this sequence as [CLS] token. By observing the visualized self-attention matrix, we can determine which frame token has a higher similarity with the classification token, which indicates that the model pays more attention to the corresponding frame when making decisions.


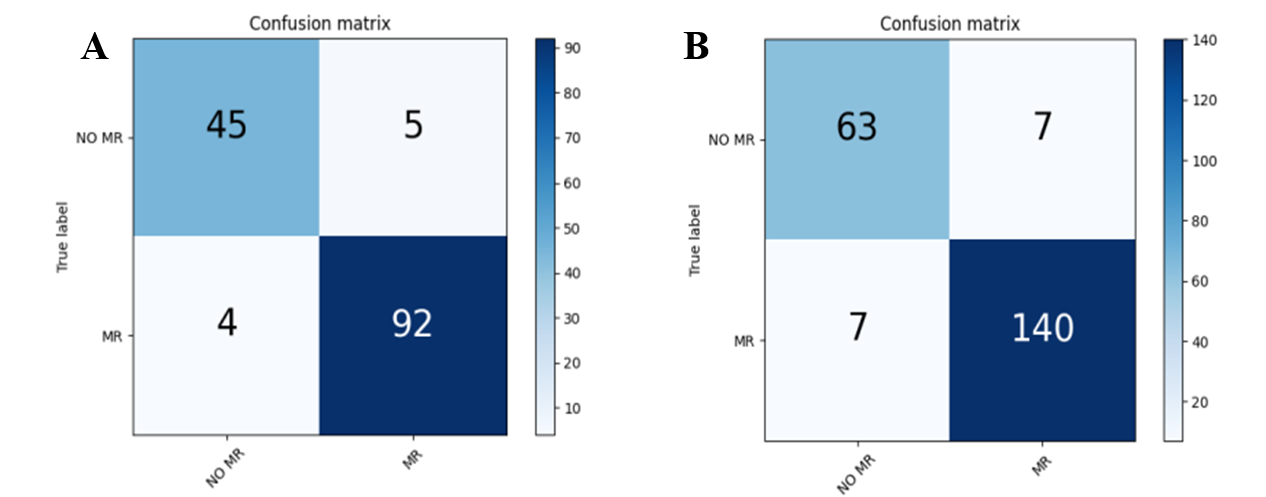


**Supplementary Figure 5** Confusion matrix diagram of the multi-view detection of MR in the retrospective and prospective test sets (A is the retrospective data set, B is the prospective data set)


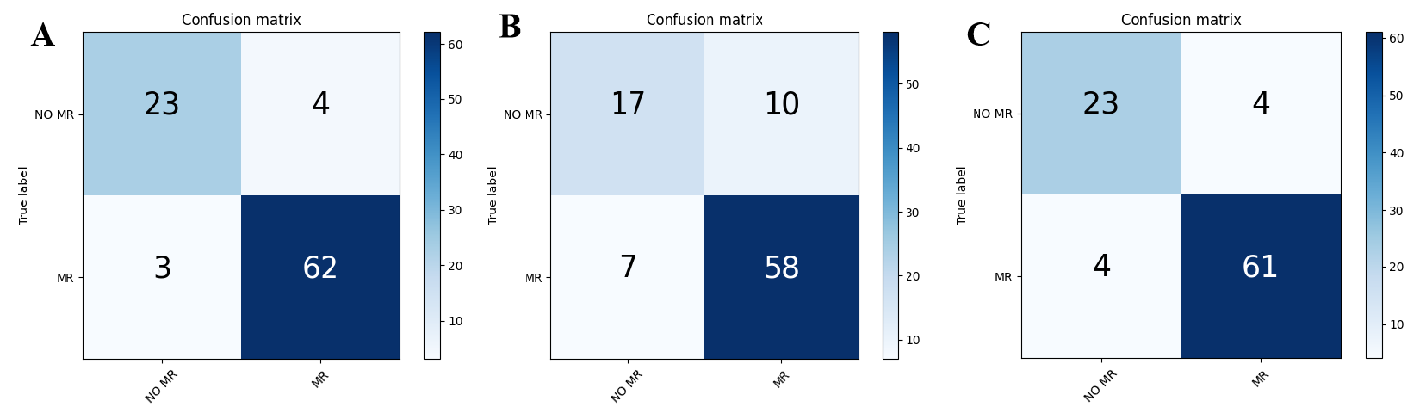


**Supplementary Figure 6** Confusion matrix diagram of the PLAX-Color, A4C-Color, and multi-view detection of MR in the retrospective test set (A: Multi-view, B: PLAX-Color, C: A4C-Color).


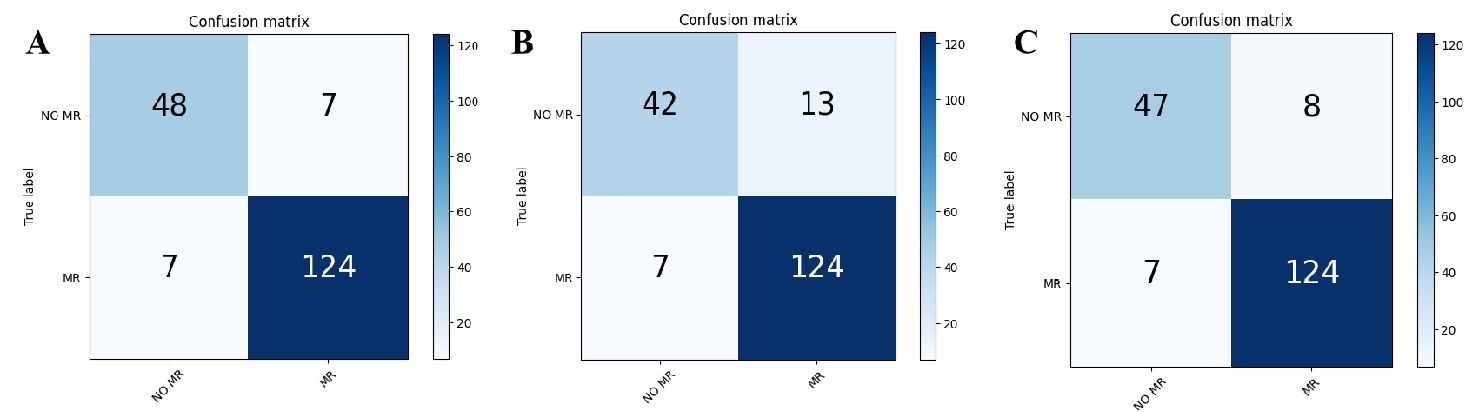


**Supplementary Figure 7** Confusion matrix diagram of the PLAX-Color, A4C-Color, and multi-view detection of MR in the prospective test set (A : Multi-view, B: PLAX-Color, C: A4C-Color).


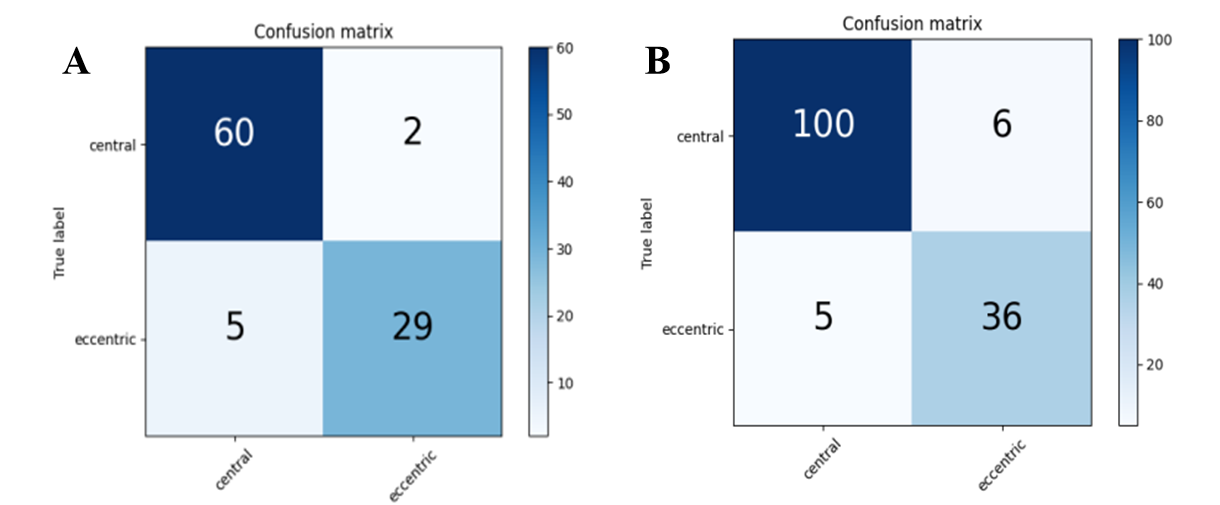


**Supplementary Figure 8** Confusion matrix for central-eccentric MR classification from multi-view model in the retrospective and prospective test sets (A is the retrospective data set, B is the prospective data set)


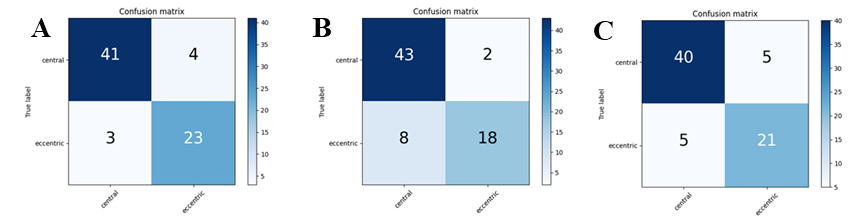


**Supplementary Figure 9** Confusion matrix diagram of the PLAX-Color, A4C-Color, and multi-view detection of central or eccentric MR in the retrospective test set (A: Multi-view, B: PLAX-Color, C: A4C-Color).


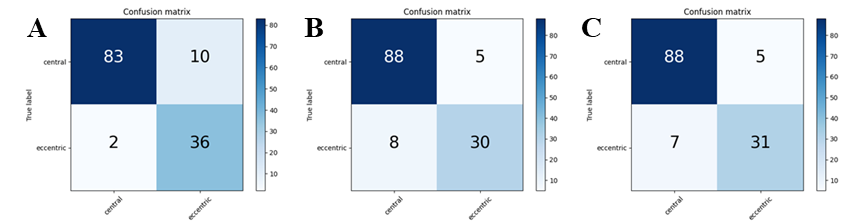


**Supplementary Figure 10** Confusion matrix diagram of the PLAX-Color, A4C-Color, and multi-view detection of central or eccentric MR in the prospective test set (A : Multi-view, B: PLAX-Color, C: A4C-Color).


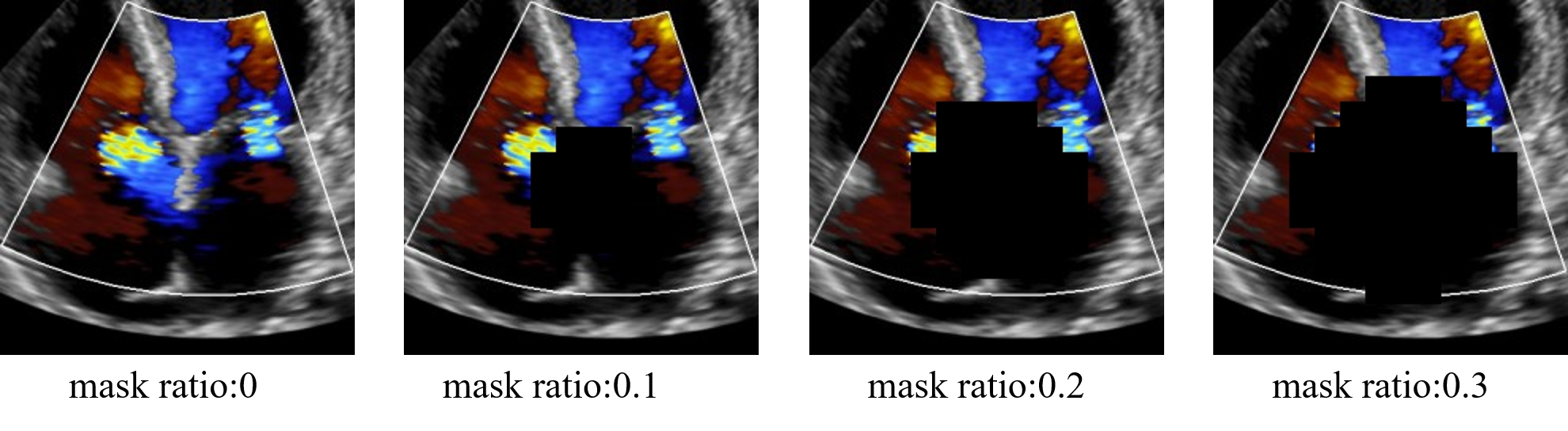


**Supplemental Figure 11**. Schematic diagram of progressive masking ablation of color Doppler region. The masks are centrally positioned, with the number of masking blocks (i.e., masking ratio) increasing sequentially from left to right.


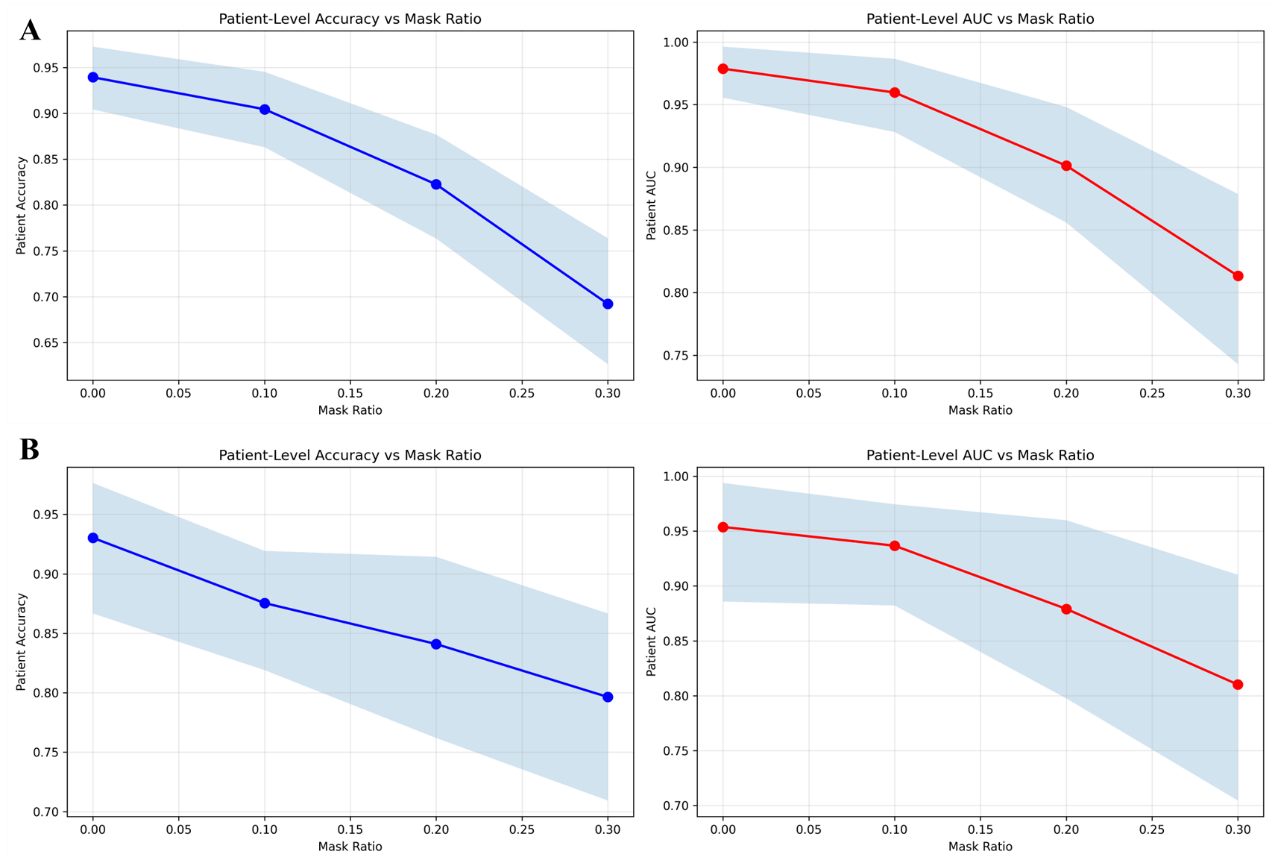


**Supplemental Figure 12.** Changes in model performance (accuracy and AUC) on the retrospective dataset with increasing masking ratio. **(A)** MR detection model. **(B)** central-eccentric MR model.


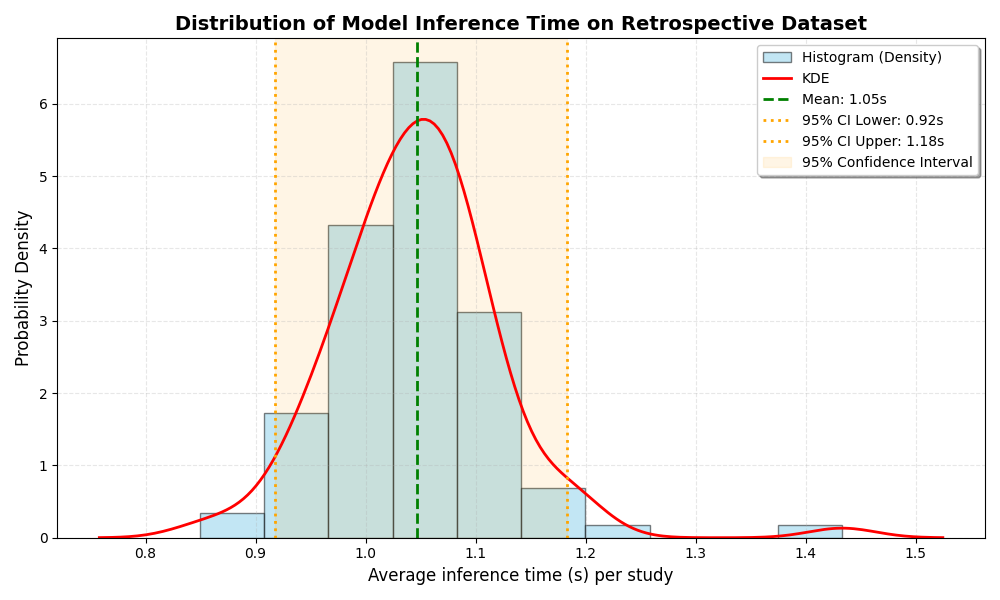


**Supplementary Figure 13**: Distribution of the average inference time per sample for the MR detection model on the retrospective test set. Note that the central-eccentric model has the same number of parameters as the MR detection model, therefore their inference times are similar.

| Supplementary Table 1. Number of videos and frames of the Retrospective Training Database | | | | | | |
| --- | --- | --- | --- | --- | --- | --- |
|  | | PLAX | A4C | A3C | A2C | A5C |
| No MR | Videos | 237 | 258 | 206 | 166 | 142 |
|  | Frames | 8982 | 12010 | 8985 | 7903 | 5903 |
| MR | Videos | 554 | 609 | 441 | 274 | 267 |
|  | Frames | 20742 | 25508 | 18839 | 12648 | 11911 |

| Supplementary Table 2. Number of videos and frames of the Retrospective Test Database | | | | | | |
| --- | --- | --- | --- | --- | --- | --- |
|  | | PLAX | A4C | A3C | A2C | A5C |
| No MR | Videos | 63 | 63 | 48 | 29 | 38 |
|  | Frames | 2624 | 2999 | 2222 | 1285 | 1752 |
| MR | Videos | 141 | 158 | 97 | 61 | 67 |
|  | Frames | 5033 | 6444 | 4040 | 2639 | 2825 |

| Supplementary Table 3. Number of videos and frames of the Prospective Test Database | | | | | | |
| --- | --- | --- | --- | --- | --- | --- |
|  | | PLAX | A4C | A3C | A2C | A5C |
| No MR | Videos | 112 | 80 | 85 | 53 | 75 |
|  | Frames | 4751 | 3894 | 4259 | 2942 | 3879 |
| MR | Videos | 292 | 256 | 236 | 97 | 213 |
|  | Frames | 11588 | 11487 | 10352 | 4500 | 9419 |

| Supplementary Table4. Distribution of central and eccentric MR in retrospective and prospective datasets | | | | |
| --- | --- | --- | --- | --- |
| Dataset | | Train set | Val set | Test set |
| retrospective | central | 234 | 38 | 62 |
|  | eccentric | 104 | 11 | 34 |
| prospective | central | - | - | 106 |
|  | eccentric | - | - | 41 |

| Supplementary Table 5. Comparison between Transformer and physician diagnostic  In Retrospective Test Database | | | |
| --- | --- | --- | --- |
| Class | Accuracy | Sensitivity | Specificity |
| Junior Doctor 1 | 0.89 | 0.98 | 0.69 |
| Junior Doctor 2 | 0.88 | 0.98 | 0.68 |
| Middle Doctor 1 | 0.92 | 0.96 | 0.84 |
| Middle Doctor 2 | 0.93 | 0.96 | 0.86 |
| Transformer | 0.94 | 0.96 | 0.9 |

Supplementary Table 6. Performance of different models in identifying MR in Retrospective test Database

| Model | Accuracy | Precision | Recall | F1-score | p-value |
| --- | --- | --- | --- | --- | --- |
| Resnet18+MIL | 0.82 | 1.00 | 0.72 | 0.84 | - |
| Resnet18+DS-MIL | 0.87 | 0.98 | 0.82 | 0.89 | <0.001 |
| ViT-B+DS-MIL | 0.89 | 0.95 | 0.88 | 0.91 | 0.04 |
| Swin-T+DS-MIL | 0.90 | 0.96 | 0.89 | 0.92 | 0.61 |
| Swin-T+DS-MIL+SSA | 0.95 | 1.00 | 0.93 | 0.96 | 0.003 |

p-values from Paired t-test comparing each row of the table with the model in the previous row.

Supplementary Table7. Performance of the model in identifying MR at the video level in the retrospective and prospective test databases

| Retrospective Test Database | | | | |
| --- | --- | --- | --- | --- |
| Class | Accuracy  (95%CI) | Precision  (95%CI) | Recall  (95%CI) | F1-score  (95%CI) |
| MR | 0.86(0.83-0.90) | 0.88(0.82-0.93) | 0.89(0.84-0.93) | 0.88(0.85-0.92) |
| NO MR |  | 0.84(0.80-0.89) | 0.83(0.76-0.89) | 0.84(0.79-0.88) |
| Prospective Test Database | | | | |
| MR | 0.90(0.88-0.92) | 0.96(0.94-0.98) | 0.89(0.85-0.92) | 0.92(0.90-0.94) |
| NO MR |  | 0.81(0.75-0.86) | 0.93(0.90-0.95) | 0.87(0.83-0.90) |

Supplementary Table 8. Performance of the model in Classifying Central or Eccentric MR at the video level in the retrospective and prospective test databases

| Retrospective Test Database | | | | |
| --- | --- | --- | --- | --- |
| Class | Accuracy  (95%CI) | Precision  (95%CI) | Recall  (95%CI) | F1-score  (95%CI) |
| Central MR | 0.88 (0.84-0.92) | 0.95(0.92-0.97) | 0.90 (0.85-0.93) | 0.92 (0.89-0.95) |
| Eccentric MR |  | 0.69 (0.59-0.79) | 0.83 (0.73-0.91) | 0.75(0.66-0.83) |
| Prospective Test Database | | | | |
| Central MR | 0.88 (0.85-0.91) | 0.95 (0.92-0.97) | 0.90(0.87-0.94) | 0.93 (0.90-0.95) |
| Eccentric MR |  | 0.65(0.55-0.74) | 0.79 (0.69-0.88) | 0.71 (0.64-0.78) |


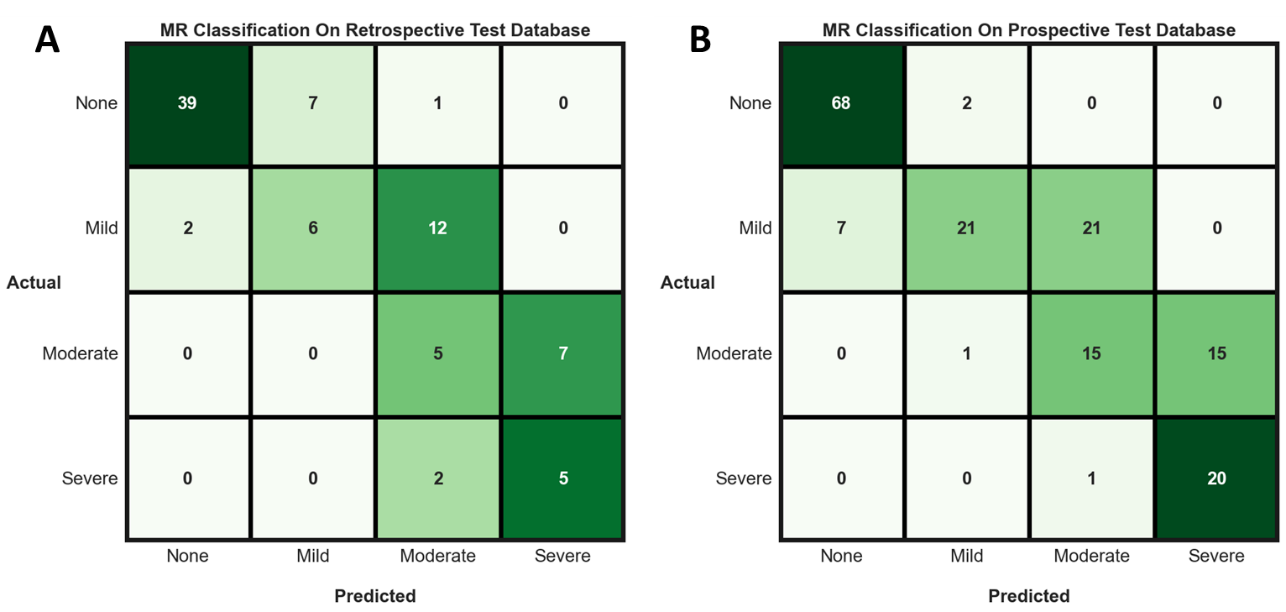


**Supplementary Figure 14**: Performance of the echonet/MR classification model on retrospective (A) and prospective (B) test sets.
